# Saturated Fat-Mediated Upregulation of IL-32 and CCL20 in Hepatocytes Contributes to Higher Expression of These Fibrosis-Driving Molecules in MASLD

**DOI:** 10.3390/ijms241713222

**Published:** 2023-08-25

**Authors:** Katharina Schilcher, Rania Dayoub, Marion Kubitza, Jakob Riepl, Kathrin Klein, Christa Buechler, Michael Melter, Thomas S. Weiss

**Affiliations:** 1 Children’s University Hospital (KUNO), University Hospital Regensburg, 93053 Regensburg, Germany; 2 Dr. Margarete Fischer-Bosch Institute of Clinical Pharmacology and University of Tuebingen, 70376 Stuttgart, Germany; 3 Department of Internal Medicine I, University Hospital Regensburg, 93053 Regensburg, Germany; 4 Center for Liver Cell Research, University Hospital Regensburg, 93053 Regensburg, Germany

**Keywords:** MASLD, NAFLD, MASH, NASH, steatosis, interleukin 32, chemokine CC ligand 20, oxidative stress, saturated fatty acid, MAPK pathway

## Abstract

Metabolic dysfunction-associated steatotic liver disease (MASLD) comprises a spectrum of liver diseases, ranging from liver steatosis to metabolic dysfunction-associated steatohepatitis (MASH), increasing the risk of developing cirrhosis and hepatocellular carcinoma (HCC). Fibrosis within MASLD is critical for disease development; therefore, the identification of fibrosis-driving factors is indispensable. We analyzed the expression of interleukin 32 (IL-32) and chemokine CC ligand 20 (CCL20), which are known to be linked with inflammation and fibrosis, and for their expression in MASLD and hepatoma cells. RT-PCR, ELISA and Western blotting analyses were performed in both human liver samples and an in vitro steatosis model. IL-32 and CCL20 mRNA expression was increased in tissues of patients with NASH compared to normal liver tissue. Stratification for patatin-like phospholipase domain-containing protein 3 (PNPLA3) status revealed significance for IL-32 only in patients with I148M (rs738409, CG/GG) carrier status. Furthermore, a positive correlation was observed between IL-32 expression and steatosis grade, and between IL-32 as well as CCL20 expression and fibrosis grade. Treatment with the saturated fatty acid palmitic acid (PA) induced mRNA and protein expression of IL-32 and CCL20 in hepatoma cells. This induction was mitigated by the substitution of PA with monounsaturated oleic acid (OA), suggesting the involvement of oxidative stress. Consequently, analysis of stress-induced signaling pathways showed the activation of Erk1/2 and p38 MAPK, which led to an enhanced expression of IL-32 and CCL20. In conclusion, cellular stress in liver epithelial cells induced by PA enhances the expression of IL-32 and CCL20, both known to trigger inflammation and fibrosis.

## 1. Introduction

A higher standard of living in Global Northern countries results in improved living conditions, but it has also led to a rise of lifestyle-associated diseases. Among others, the prevalence of obesity and associated diseases such as type 2 diabetes and further manifestations of the metabolic syndrome are severely increasing [1]. Metabolic dysfunction-associated steatotic liver disease (MASLD) [2], formerly termed as non-alcoholic fatty liver disease (NAFLD), which has a global prevalence of 30%, is the hepatic manifestation of the metabolic syndrome and has therefore become the leading cause of chronic liver diseases worldwide [3]. MASLD is caused by the abnormal or excessive accumulation of lipid droplets in hepatocytes [1]. It covers a range of liver disorders encompassing simple steatosis, metabolic dysfunction-associated steatohepatitis (MASH) [2], formerly termed as non-alcoholic steatohepatitis (NASH), and its progression toward hepatic cirrhosis [3]. Numerous studies have analyzed differential gene and protein expression in MASLD; however, the precise underlying mechanisms of the pathogenesis of MASH in the steatotic liver have just begun to be understood [4,5,6].

According to the multiple-hit hypothesis of development and progression of MASLD, steatosis, lipo-toxicity and inflammation play an important role [4]. Hepatic steatosis, especially the accumulation of saturated free fatty acids (FFAs) such as palmitic acid (PA) and cholesterol, leads to lipo-toxicity, which causes a change in behavior of liver cells. It is mediated via various pathways including the modification of mitochondrial function, oxidative stress and the activation of signaling pathways and death receptors [5]. Thereby, transcription factors, such as NF-kB, are activated, resulting in enhanced expression and release of pro-inflammatory mediators like tumor necrosis factor (TNF) and interleukins [4,5]. TNF mediates liver damage by inducing the production of reactive oxygen species (ROS), which in turn triggers inflammation and fibrogenesis [5]. Steatosis, MASH and early liver fibrosis are reversible manifestations of MASLD. However, if fibrogenesis progresses, the disease may proceed to irreversible forms such as advanced fibrosis, cirrhosis or hepatocellular carcinoma (HCC) [7]. Thus, if the state of fibrosis is defining for the prognosis and mortality of MASLD patients, it is indispensable to identify the driving factors of fibrogenesis [8].

MASH is a multifactorial liver disorder and knowledge about the impact of each factor driving disease progression is limited. Inflammatory and fibrogenic signaling events play major roles for the pathogenesis of MASLD and its progression [6]. In a preliminary study, we identified differentially expressed genes (DEGs) in liver samples of patients with MASH such as interleukin-32 (IL-32) and chemokine CC ligand 20 (CCL20), which are associated with fibrogenesis and MASLD. IL-32 is known to be the pivotal regulator of liver inflammation caused by obesity [9]. CCL20, also called macrophage inflammatory protein-3alpha, contributes to chronic liver inflammation and fibrosis by mediating the chemotaxis of immune cells [10]. Less is known about the molecular mechanism of their induction under conditions of MASLD. Fatty acids, in particular saturated fatty acids, play an important role in the pathogenesis of MASLD and have been shown to induce lipo-toxicity, resulting in the expression of genes induced by stress [4,5,11]. Furthermore, a predisposing pro-steatotic genetic risk factor is patatin-like phospholipase domain-containing protein 3 (PNPLA3 or adiponutrin), which is highly expressed in liver tissue. PNPLA3 is a lipid droplet-associated protein with hydrolase activity for triglycerides and retinyl esters. Its I148M variant is known to be involved in the pathogenesis of MASLD. By impairing the metabolism of lipid droplets and inducing the expression of cytokines and chemokines, the PNPLA3 I148M variant contributes to liver inflammation, fibrosis and cirrhosis [12]. Consequently, we analyzed the gene expression of IL-32 and CCL20 in the liver tissue of controls and patients as well as the effect of fatty acids on their expression in hepatoma cells. Furthermore, we analyzed the impact of the PNPLA3 I148M variant on the expression of the aforementioned genes.

In the current study, we show that these pro-inflammatory factors are linked to the progression of MASH and fibrosis in human liver samples. In vitro experiments demonstrate that the expression of IL-32 and CCL20 can be induced by PA, a saturated fatty acid and well-known risk factor for lipo-toxicity [4,5,11]. Furthermore, we show that a high expression of IL-32 and CCL20 caused by saturated FFAs can be mitigated by oleic acid (OA), which is an unsaturated FFA. Finally, we identify some of the underlying signaling pathways that induce the expression of IL-32 and CCL20 in liver cells.

## 2. Results

### 2.1. IL-32 and CCL20 mRNA Expression Is Enhanced in MASLD

**Figure 1:**
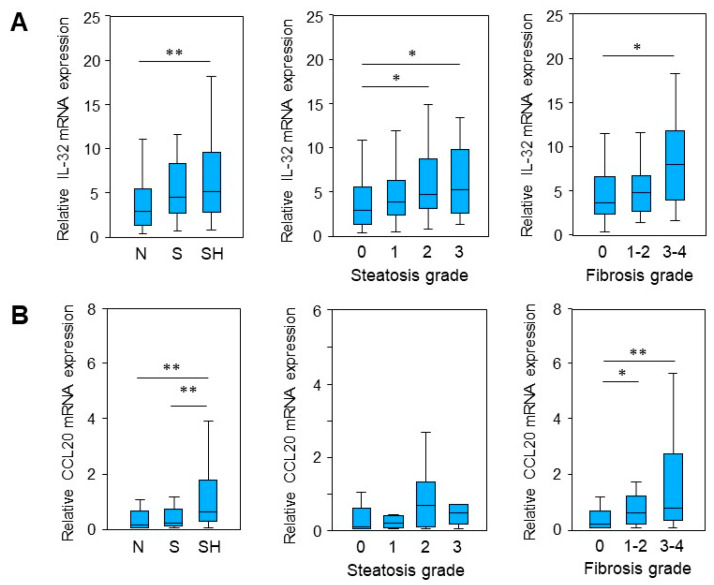
mRNA expression of IL-32 and CCL20 in liver samples from patients with MASLD. (**A**) IL-32 and (**B**) CCL20 mRNA expression was analyzed in liver tissue samples from patients with MASH (SH, n = 43), hepatic steatosis (S, n = 46) and normal liver tissue (N, n = 32) by qRT-PCR. Expression levels were plotted regarding their histologically proven steatosis grade (0 = 32, 1 = 25, 2 = 44, 3 = 21) and fibrosis grade (0 = 76, 1–2 = 27, 3–4 = 16) (for sample characteristics see Table S1). YWHAZ mRNA expression was determined for normalization. Statistical differences were analyzed by Kruskal–Wallis test with post hoc Bonferroni correction. * *p* < 0.05, ** *p* < 0.01.

In a preliminary micro-array study [13], mRNA expression in liver tissue was compared between a control group (N or normal liver), patients with steatosis (S) and a cohort suffering from MASH (SH). Differentially expressed genes (DEGs) that are involved in liver injury and tissue repair/regeneration were identified. In this study, these findings were validated in a larger cohort (n = 121) using quantitative real-time PCR (qRT-PCR) analysis. By comparison of the relative mRNA expression of the three groups, N, S and SH, we confirmed a significant upregulation in MASH patients of genes involved in liver injury and regeneration, namely Interleukin-32 (IL-32) [14] and chemokine CC ligand 20 (CCL20) [15] (Figure 1A,B). Additionally, aldo-keto reductase family 1 member B10 (AKR1B10), which is a known biomarker for steatohepatitis [16], was significantly enhanced (Supplementary Figure S1A). Furthermore, the upregulation of IL-32 expression was observed in liver samples in parallel with histological steatosis grade (Figure 1A). Moreover, IL-32 and CCL20 levels were increased in tissue samples with more progressed fibrosis (Figure 1A,B).

Adiponutrin (PNPLA3) is a lipid droplet-associated lipogenic and lipolytic enzyme, which is regulated by carbohydrates. Its common protein variant I148M (rs738409) represents the most important pro-steatotic genetic risk factor and is consequently a marker for fibrosis, cirrhosis and hepatocellular carcinoma [12]. We analyzed our sample cohort for the presence of PNPLA3 (I148M) variant carriers and identified a correlation between the variant carrier (CG/GG) with steatosis and steatohepatitis as well as with steatosis grade (Supplementary Figure S2). Stratification for the PNPLA3 I148M variant revealed significantly increased IL-32 mRNA expression in steatotic and MASH samples solely in PNPLA3 I148M (CG/GG) variant carriers, not in non-carriers (CC) (Supplementary Figure S3A). In contrast to this, significantly increased CCL20 mRNA expression in MASH samples was irrespective of PNPLA3 I148M variant status (Supplementary Figure S3B). Interestingly, a positive correlation between steatosis grade and the expression of CCL20 was observed in non-carriers of the I148M variant (Supplementary Figure S3B). Furthermore, there was no significant change in IL-32 expression observed, regarding fibrosis scores and PNPLA3 status. Contrary to this, CCL20 was differentially expressed; however, it was independent of the carrier status (Supplementary Figure S3A,B). In summary, hepatic IL-32 and CCL20 expression is increased in MASH samples with progressed activity (intense steatosis and moderate-to-severe fibrosis), and enhanced IL-32 expression was found to be related to the PNPLA3 I148M variant.

### 2.2. Treatment with Palmitic Acid Leads to Fat Deposition and Induction of IL-32 and CCL20 Expression

**Figure 2:**
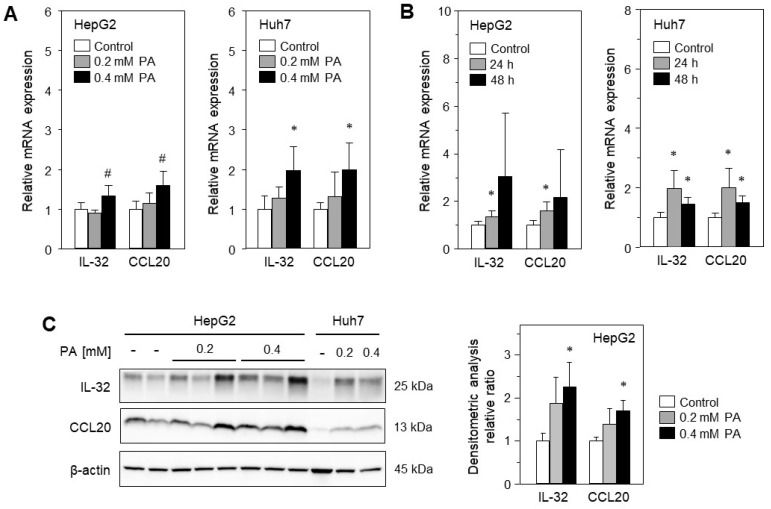
Palmitic acid (PA) induces mRNA expression of IL-32 and CCL20 in vitro. (**A**) HepG2 and Huh7 cells were treated without (control) or with indicated concentrations of PA for 24 h. (**B**) HepG2 and Huh7 cells were treated with 0.4 mM PA for indicated times (control, no treatment). The mRNA levels were analyzed and normalized to YWHAZ (three independent experiments, mean ± SD). * *p* < 0.05 differs from control, # *p* < 0.05 differs from control and 0.2 mM PA. (**C**) HepG2 and Huh7 cells were incubated without (-) or with indicated concentrations of PA for 24 h followed by isolation of total protein extracts. Western blot analysis using specific anti-IL-32 and anti-CCL20 antibodies was performed with β-actin as loading control. Western blots from HepG2 cell experiments were analyzed for relative protein abundance by densitometric analysis and normalized to loading control and untreated cells (control). Data presented as mean ± SEM, * *p* < 0.05 differs from control.

Cellular lipid accumulation is a major hallmark of MASLD, and therefore in vitro models of steatosis, based on primary hepatocytes or hepatoma cell lines, are useful to analyze fatty acid-associated cell stress and damage [17]. To shed light on the role of cellular steatosis for the expression of IL-32 and CCL20 in MASLD, we treated two hepatoma cell lines, HepG2 and Huh7, with palmitic acid (PA, C16:0), known to induce cellular lipid accumulation, lipo-apoptosis and lipo-toxicity [18]. After 24 h of treatment with PA, distinct intracellular lipid droplets were observed in both cell lines, as reported earlier [18]. They increased in a positive correlation with higher PA concentrations (0, 0.2 and 0.4 mM) and longer treatment (24 and 48 h). We analyzed the mRNA expression of IL-32 and CCL20 in the aforementioned treated cells and found enhanced expression of both genes using 0.4 mM PA for 24 h (Figure 2A) or for up to 48 h of treatment (Figure 2B). We confirmed these findings for protein expression, showing increased IL-32 and CCL20 protein levels by Western blotting (Figure 2C). Thus, we were able to show that treatment with the saturated FFA PA can induce the expression of IL-32 and CCL20, which are known to be involved in inflammation and fibrogenesis and are therefore important for liver injury.

### 2.3. Monounsaturated Oleic Acid Compared to Palmitic Acid Does Not Induce IL-32 and CCL20 Expression

**Figure 3:**
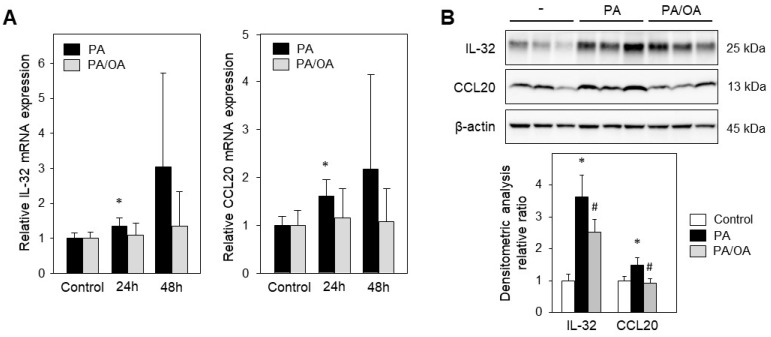
Unsaturated oleic acid (OA) reduces PA-induced expression of IL-32 and CCL20 in vitro. (**A**) HepG2 cells were incubated either with 0.4 mM PA or with 0.4 mM PA/OA (1/2) for indicated times (control, no treatment). The mRNA levels were analyzed and normalized to YWHAZ (three independent experiments, mean ± SD). * *p* < 0.05 differs from control. (**B**) HepG2 cells were incubated without PA, with 0.4 mM PA or 0.4 mM PA/OA (1/2) for 24 h followed by isolation of total protein extracts. Western blot analysis using specific anti-IL-32 and anti-CCL20 antibodies was performed with β-actin as loading control. Immunoblots were analyzed by densitometry and normalized to loading control and untreated cells (control). Data presented as mean ± SEM, * *p* < 0.05 differs from control, # *p* < 0.05 differs from PA treatment.

Monounsaturated fatty acids have been shown to attenuate PA-mediated lipo-apoptosis and cyto-toxicity [19,20]. Therefore, we analyzed the impact of oleic acid, OA (C18:1), on the MASH-related genes IL-32 and CCL20. HepG2 cells incubated with 0.4 mM PA for 24 h showed a significant upregulation of IL-32 and CCL20 (Figure 3A,B). Treating the cells with a mixture of 0.4 mM PA/OA (1/2) did not result in increased mRNA expression of the analyzed genes (Figure 3A). Western blotting revealed a reduction in IL-32 and CCL20 protein levels upon addition of OA (PA/OA, mixture 1/2) compared to PA alone (0.4 mM) (Figure 3B). Therefore, the presented data highlight the protective role of OA by reducing the expression of factors known to play a key role in inflammation and fibrogenesis.

### 2.4. Palmitic Acid-Induced Gene Expression Is Mediated via Stress-Induced Pathways

**Figure 4:**
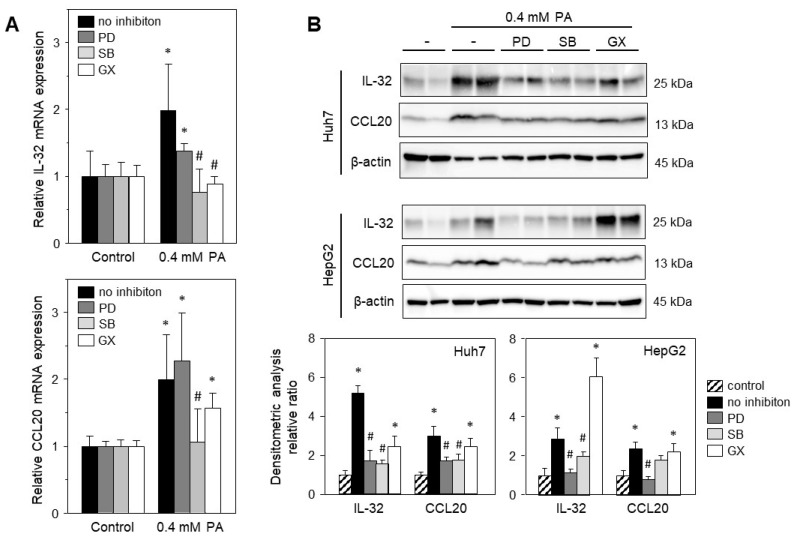
PA-induced expression of IL-32 and CCL20 is mediated by Erk1/2 and p38 MAPK. (**A**) Huh7 cells were treated with 0.4 mM PA for 24 h, and without (no inhibition) or with (1.5 h prior to PA) addition of specific inhibitors: PD098059 (PD, 10 µM) for Erk1/2, SB203580 (SB, 10 µM) for p38 MAPK or GF109203X (GX, 10 µM) for PKC signaling. The mRNA levels were analyzed and normalized to YWHAZ (three independent experiments, mean ± SD). * *p* < 0.05 differs from control, # *p* < 0.05 differs from no inhibition. (**B**) Huh7 and HepG2 cells were treated without or with 0.4 mM PA for 24 h, and with or without PD, SB or GX as described above (**A**) followed by isolation of total protein extracts. Western blot analysis using specific anti-IL-32 and anti-CCL20 antibodies was performed with β-actin as loading control. Immunoblots were analyzed by densitometry and normalized to loading control and untreated cells (control). Data presented as mean ± SEM, * *p* < 0.05 differs from control, # *p* < 0.05 differs from no inhibition.

FFAs have been shown to induce cell stress and thereby mediate the expression of a variety of genes. To elucidate the signaling pathways induced by PA, we treated HepG2 or Huh7 cells with PA and specific inhibitors of the PKC and MAPK (Erk1/2 and p38) pathways. PA-induced expression of IL-32 and CCL20 was significantly reduced by inhibition of the Erk1/2 and p38 pathways (Figure 4B). Notably, PA-induced IL-32 and CCL20 mRNA expression was diminished by the inhibition of the p38 but not of the Erk1/2 pathway in Huh7 cells (Figure 4A). Inhibition of PKC did not change CCL20 mRNA (Figure 4A) or protein expression (Figure 4B). IL-32 protein expression was not reduced by PKC blockade in both cell lines (Figure 4B), although mRNA expression was reduced in Huh7 cells (Figure 4A). Overall, the PA-induced expression of IL-32 and CCL20 is mainly mediated by Erk1/2 and p38 MAPK.

## 3. Discussion

In the present study, we analyzed the expression of liver injury-related genes in patients with MASLD and could demonstrate a significant upregulation of IL-32 and CCL20 in MASH samples with progressed activity (intense steatosis and moderate-to-severe fibrosis). The in vitro experiments involved treating hepatoma cells with FFAs to induce steatosis and lipo-toxicity, both of which are known to correlate with the severity of MASLD. Gene and protein expression was induced by PA, and this PA-mediated upregulation was attenuated by OA. Finally, we were able to provide evidence and shed light on the molecular mechanisms and signaling pathways involved in the differential expression of these genes.

Elevated serum levels of FFAs have been shown to be associated with the progression of MASLD [21]. In addition, FFAs and their metabolites have been proven to be important mediators of lipo-toxicity (including ROS-stress), leading to hepatocellular injury and the progression of MASLD [21]. While the overabundance of saturated fatty acids, such as PA, increases the lipo-toxicity, and therefore lipo-apoptosis and the activation of ER-stress response pathways in liver cells [18], unsaturated fatty acids mitigate these effects. Monounsaturated OA has been shown to be less lipo-toxic and abate the saturated (e.g., PA) FFA-induced lipo-toxicity by inhibiting ER-stress in hepatic cells [19,22,23]. The induction of ER- and ROS-stress by PA activates signaling pathways such as PKC, JNK and MAPK (Erk1/2, p38), resulting in the translocation of transcription factors NFκB, AP1 and SP1, which are well-recognized for their importance in inflammation [24,25].

IL-32 has been initially described as a pro-inflammatory cytokine, which is expressed by immune cells such as monocytes, natural killer (NK) and T-cells, as well as by various non-immune cells including endothelial and epithelial cells [26]. IL-32, when secreted, can induce inflammatory cytokines including TNF, IL-6 and IL-1*β* as well as macrophage inflammatory protein-2 (MIP-2) and is associated with several diseases and inflammatory conditions [26]. Elevated IL-32 serum levels have been reported in chronic obstructive pulmonary disease [27], type 2 diabetes [28], HIV infection [29] and MASLD [9]. Although the secretory pathway of IL-32 and its cell surface receptor for signal transduction is still not completely understood, it has been reported that overexpressed or induced IL-32 is not secreted from hepatoma cells or hepatocytes infected with hepatitis B virus (HBV) [30]. Correspondingly, in our in vitro experiments we analyzed IL-32 in cell culture supernatant using ELISA and only found levels below the detection limit of the assay, irrespective of treatment with PA.

The expression of IL-32 is upregulated by several cytokines, including TNF and IL-1β, as well as by infection, pathogen-associated molecular patterns (PAMPs) and oxidative stress [26]. Hypoxia-induced ROS have been shown to increase IL-32 expression in breast cancer cells [31]. Furthermore, is has been reported that the stability of the IL-32 protein is regulated by the deoxygenation of the N-terminal cysteine by a thiol oxidase [32], which highlights the role of oxygen-sensing systems in regulating IL-32 expression in response to oxidative stress. Consistent with this, we have shown increased IL-32 expression in hepatoma cells upon treatment with PA, known to generate ROS, which is mitigated by OA [22,23]. As reported elsewhere [9,33], in this study we found enhanced IL-32 expression in the liver tissue of patients with MASH. This may be caused by ROS-stress, which is induced by the accumulation of saturated fat in hepatocytes [21]. In addition, we present data that indicate the involvement of activated MAPK (Erk1/2, p38) cascades in IL-32 expression, which has been shown to be stimulated by ROS-stress [34] and further points to the important role of ROS in IL-32 regulation under steatotic conditions. The activation of these signaling pathways may lead to nuclear translocation of NFκB, c-Jun (AP1) and SP1 and subsequently to the activation of their respective target genes. The IL-32 promoter harbors an SP1 binding site, as well as contains binding sites for fatty acid-sensitive transcription factors, such as PPARγC1α and RXRα, indicating a potential direct impact of FFAs on IL-32 expression [9].

Beside the effect of secreted IL-32 on cytokine expression, and consequently on the induction of inflammation, cellular IL-32 expression shows a variety of molecular interventions. Increased IL-32 expression has been found in hepatocytes of patients suffering from chronic HBV [30] or HCV [35] infection. Interestingly, the overexpression of IL-32 in hepatoma cells in vitro revealed a potent antiviral effect against HBV [36], but on the other hand had no influence on HCV virus replication in [35]. Damen et al. reported a role of IL-32 in hepatic cholesterol homeostasis by regulating the lipid regulatory receptor LXRα, lipid transporters ABCA1 and ABCG1 as well as the fatty acid carrier ApoA1 in liver cells, which is followed by a reduction in intracellular lipid levels [37]. In addition, increased IL-32β expression has been reported to decrease intracellular lipid concentrations in hepatoma cells, which may be mediated by reduced PPARγ expression and elevated AMPK activity [38]. In summary, increased IL-32 expression under MASLD conditions or upon treatment with FFAs may be a cellular response to minimize lipo-toxicity. Furthermore, IL-32 levels have been reported to be associated with obesity-related inflammation [39] and a correlation with disease severity in MASLD has been observed [9]. In agreement with this study, we observed a positive correlation between hepatic IL-32 expression and steatosis as well as fibrosis grade in the MASLD patients, albeit only a weak correlation with inflammation and obesity (Supplementary Table S2).

Stratification by PNPLA3 gene variant status, which has been shown to influence liver disease from simple steatosis to MASH, fibrosis and hepatocellular carcinoma [12], revealed a clear correlation between IL-32 and disease severity of MASH in PNPLA3 variant carriers, which is in agreement with a report by Baselli et al. [9]. Moreover, IL-32 expression and PNPLA3 gene variant were not found to be dependent on fibrosis stage in our cohort, contrary to previous reports [9]. The observed discrepancies in the results could be due to a difference in study population and design; for example, we performed RT-PCR compared to gene array technology [9]. However, as a common conclusion as well as validation of our study, we and other similar studies found a significant increase in AKR1B10 expression in patients with steatohepatitis [9,16]. Furthermore, AKR1B10 did not correlate with steatosis grade, but did significantly with fibrosis score (Supplementary Figure S1B,C), indicating that AKR1B10 is a marker of MASH progression. Additionally, it has been shown that AKR1B10 is upregulated by oxidative and ER-stress, and that the PNPLA3 gene variant did not control discrete AKR1B10 expression [40], which is in line with our results (Supplementary Figure S1).

CCL20 is a pro-inflammatory chemokine, which can be induced in a variety of cells by lipopolysaccharide, TNF and IL-1β, and consequently attracts chemokine receptor 6 positive cells. CCL20 has been described to substantially augment inflammation and fibrogenesis in patients suffering from alcoholic hepatitis [41]. Furthermore, it was reported that CCL20 is upregulated in hepatocytes upon injury and improves hepatic fibrosis by recruiting γδT cells in CCl_4_ mouse models [10]. Upregulated CCL20 expression has been observed under conditions of hepatic fibrosis in patients with chronic liver diseases, such as hepatitis C virus infection, alcoholic steatohepatitis or primary biliary cirrhosis [10] and in patients with MASH [42]. The data presented in our study are consistent with these reports by indicating a significant increase in CCL20 expression in MASH livers, irrespective of PNPLA3 carrier status. Additionally, CCL20 expression did not correlate with steatosis grade, but with fibrosis stage and tissue inflammation status (inflammatory cells). This supports the role of CCL20 in the pathogenesis of fibrosis and hepatic inflammation, while being independent of the PNPLA3 carrier group (Supplementary Table S2). Contradictory data have been reported regarding CCL20 mRNA induction by FFAs. Some show no effect of lipid loading with PA or OA treatment [40], while others indicate increased CCL20 expression in HepG2 cells in response to PA [42]. Our data show enhanced CCL20 mRNA and protein expression in HepG2 and Huh7 cells induced by saturated PA, which is diminished by the addition of monounsaturated OA and aligns our IL-32 data. Interestingly, Baselli et al. found CCL20 mRNA expression to be co-regulated with IL-32 in livers from patients with severe obesity [9], suggesting a common regulatory mode. As previously described for IL-32, oxidative stress caused by PA treatment, TNF and IL-1β, induces CCL20 expression via activation of MAPK (Erk1/2, p38) pathways and the nuclear translocation of NFκB, c-Jun (AP1) and SP1 [34]. The CCL20 promoter harbors binding sites for NFkB, AP1 and SP1 [43], hence making it most likely to be induced by ROS.

MASLD encompasses a continuous spectrum of liver diseases, ranging from non-alcoholic fatty liver to MASH, which increases the risk of developing fibrosis, cirrhosis and HCC [3]. Changes in the expression patterns of genes associated with liver regeneration have been identified in steatotic livers, leading to compromised functional recovery after tissue loss [44]. Here, we present data showing increased expression of IL-32 and CCL20 in patients with MASH, as well as their induction by PA in hepatoma cells, likely mediated by oxidative stress and cellular lipid loading. This enhanced expression may represent a dichotomous result, indicating on one hand a response to liver injury, which consequently triggers inflammation and wound healing (fibrogenesis), but on the other hand also an attempt to maintain metabolic homeostasis. These factors are important for inflammation and fibrosis and must therefore be evaluated for potential therapeutic interventions.

## 4. Materials and Methods

*Study Subjects and Collection of Samples:* Human liver tissues samples of patients without MASLD (n = 32), patients with simple liver steatosis (n = 46) and patients with MASH (n = 43) were examined and analyzed as described previously [13] (for tissue characteristics see Supplementary Table S1). The experimental procedures were performed according to the guidelines of the charitable state-controlled foundation HTCR (Human Tissue and Cell Research, Regensburg, Germany), with written informed consent from patients. The study and the consent form were approved by the local ethical committee of the University of Regensburg (ethics statement 12-101-0048, University of Regensburg, Germany). All experiments involving human tissues and cells have been carried out in accordance with *The Code of Ethics of the World Medical Association* (Declaration of Helsinki).

*Cell culture and treatments:* The human hepatoma cell line HepG2 was obtained from American Type Culture Collection (HB-8065, ATCC, Manassas, VA, USA) and Huh7 cells (ECACC 01042712) from European Collection of Authenticated Cell Cultures (ECACC) (Salisbury, UK). Cells were grown at 37 °C, 5% CO_2_ in DMEM (BioWhittaker, Verviers, Belgium) supplemented with penicillin (100 units/mL), streptomycin (10 μg/mL) and 10% fetal calf serum (Biochrom, Berlin, Germany). Cells were seeded at a density of 5 × 10^4^ cells/cm^2^, in 6-well plates, cultivated for 24 h, and after an additional 24 h of starvation (serum free culture medium DMEM) the cells were treated with indicated concentrations of either palmitic acid (PA) or with a mixture of palmitic and oleic acid (ratio 1:2) (PA/OA) for 24 or 48 h. Free fatty acids (OA, #01008-5G, PA #P0500-10G) were obtained from Sigma-Aldrich (Deisenhofen, Germany) and dissolved in isopropanol. Specific inhibitors (10 µM each) were added 1.5 h before application of PA for blocking signaling pathways known to be activated by FFAs: PD098059 (#P215-1MG; Erk1/2 inhibitor) and SB203580 (#S8307-1MG; p38 MAPK inhibitor), purchased from Sigma-Aldrich, as well as GF109203X (#0741; protein kinase C inhibitor) obtained from Tocris Bio-Techne GmbH (Wiesbaden-Nordenstadt, Germany), were dissolved in DMSO.

*RNA isolation, reverse transcription, qRT-PCR and genotyping:* Total RNA was isolated using RNeasy Mini Kit (Qiagen, Hilden, Germany). One μg of total RNA was reverse-transcribed using the Reverse-Transcription System (Qiagen, Hilden, Germany). Following primers were used (Metabion, Martinsried, Germany): IL-32 Fwd.: 5′-tcaaagagggctacctggag-3′, IL-32 Rev.: 5′-tttcaagtagaggagtgagctctg-3′. CCL20 Fwd.: 5′-ctggctgctttgatgtcagtgct-3′, CCL20 Rev.: 5′-gcagtcaaagttgcttgctgcttc-3′. YWHAZ Fwd.: 5′-gcaattactgagagacaacttgaca-3′, YWHAZ Rev.: 5′-tggaaggccggttaatttt-3′. Transcript levels were quantified using real-time PCR technology (Roche, Penzberg, Germany). PCR products were verified by sequence analysis and each quantitative PCR was performed in triplicates. Genotyping for rs738409C>G in PNPLA3 gene was performed using genomic DNA and a predeveloped assay C_7241_10 on a TaqMan 7900HT device (Thermo Fischer Scientific, Dreieich, Germany) according to manufacturer’s instructions. Genotype results did not deviate from Hardy–Weinberg equilibrium.

*SDS-PAGE and immunoblotting:* Total proteins were isolated and subjected to Western blot analysis as described [45]. Briefly, 30 μg protein per lane were separated by 12% SDS-PAGE (Biorad, Hercules, CA, USA) under reducing conditions, and proteins were transferred onto PVDF membranes (Biorad, Hercules, CA, USA), incubated with specific antibodies and developed using enhanced chemiluminescence reagent (Thermo Fisher Scientific, Darmstadt, Germany). The following antibodies were used: IL-32 (ab172339) was obtained from Abcam (Cambridge, UK), CCL20 (LS-C104608) from BIOZOL Diagnostica (Eching, Germany) and ß-actin (#4970) was obtained from Cell Signaling (Danvers, MA, USA). Secondary goat HRP-conjugated antibodies (anti-rabbit #P0448 and anti-mouse #P0447) were obtained from Dako (Hamburg, Germany).

*Statistical analysis:* mRNA expression results of patient samples were evaluated for normality distribution by a Shapiro–Wilk test. Data presented as box plots displaying median values, lower and upper quartiles and the range of the values. Statistical differences between two groups were analyzed by a two-tailed Mann–Whitney U Test or a Student’s unpaired *t*-test (in vitro) and between several groups (data from human samples) by a Kruskal–Wallis Test with post hoc Bonferroni correction where appropriate. Values of *p* < 0.05 were considered significant (SPSS Statistics 25.0 program, IBM, Leibniz Rechenzentrum, München, Germany). Each experiment was performed at least in triplicates and results were expressed as means ± SD (standard deviation) or SEM (standard error of the mean) as indicated.

## Data Availability

All relevant data are within the paper.

## Supplementary Materials

The following supporting information can be downloaded at: https://www.mdpi.com/article/10.3390/ijms241713222/s1.
