# Supplementary material for: Saturated Fat-Mediated Upregulation of IL-32 and CCL20 in Hepatocytes Contributes to Higher Expression of These Fibrosis-Driving Molecules in MASLD
Source: Int J Mol Sci. 2023 Aug 25;24(17):13222. doi: 10.3390/ijms241713222 (PMC10487578; doi:10.3390/ijms241713222)
Supplement: Supplementary file 1 [file ijms-24-13222-s001.zip › ijms-2552458-supplementary.pdf]

Saturated fat mediated upregulation of IL-32 and CCL20 in hepatocytes contributes to higher expression of these fibrosis driving molecules in MASLD

Katharina Schilcher[1], Rania Dayoub[1], Marion Kubitza[1], Jakob Riepl[1], Kathrin Klein[2], Christa Buechler[3], Michael Melter[1], Thomas S. Weiss[1,4]

Supplementary Table

Table S1. Age, body mass index (BMI), steatosis, inflammation- and fibrosis scores of the cohort studied for gene expression analysis as described earlier [1]. Data are shown as count for patients and PNPLA3 or median (range of values) for age and BMI.

	Normal liver (N)	Steatosis (S)	MASH (SH)
Patients (male/female)	32 (17/15)	46 (25/21)	43 (18/25)
Age [y]	58 (20-82)	60 (24-84)	65 (33-82)
BMI [kg/m^2]	25 (18-30)	29 (21-46)	28 (21-58)
Steatosis [a]	0	2 (1-3)	2 (1-3)
Inflammation [b]	0	0 (0-2)	2 (1-3)
Fibrosis [c]	0	0 (0-4)	2 (1-3)
Ballooning [d]	0	0 (0-1)	2 (1-2)
MASH activity score [e]	0 (0)	1 (1-2)	3 (3-4)
PNPLA3: CC/(CG/GG)	21/(10/1)	16/(27/3)	16/(19/5)

Human liver tissues for mRNA expression analysis were histologically examined for patients without MASLD, patients with simple liver steatosis and patients with MASH as described earlier [2]. Anamnesis excluded alcohol intake, drugs and viral infections as cause for MASLD. Surgery was done because of hepatic metastases of extrahepatic tumors (65 patients), focal nodular hyperplasia of the liver (9 patients), adenoma (7 patients), cholangiocarcinoma (12 patients), hepatocellular carcinoma (11 patients) and other diseases (6 patients) and only non-affected tissue was used. In particular, liver samples without pathological findings (without MASLD, normal liver) were obtained from resected tissue of patients undergoing surgery because of hepatic metastases of extrahepatic tumors (23 patients), focal nodular hyperplasia of the liver (4 patients), cholangiocarcinoma (4 patients) and adenoma (1 patients).

[a] Steatosis was scored as <5% steatosis (0), 5% to 33% steatosis (1), >33% to 66% steatosis (2) and >66% steatosis (3).

[b] Inflammation was scored as no foci / 20 × field (0), <2 foci / 20 × field (1), 2–4 foci / 20 × field (2) and >4 foci / 20 × field (3).

[c] Fibrosis was defined as no fibrosis (0), zone 3 perisinusoidal/ pericellular fibrosis; focally or extensively present (1), zone 3 perisinusoidal/ pericellular fibrosis with focal

or extensive periportal fibrosis (2), zone 3 perisinusoidal/ pericellular fibrosis and portal fibrosis with focal or extensive bridging fibrosis (3) and cirrhosis (4).

[d] Ballooning was scored by the number of enlarged hepatocytes as none / field (0), few ballooned hepatocytes / field (1), many ballooned hepatocytes / field (2).

[e] MASH activity (from 0 to 8) was calculated and assigned to MASH activity scores (MAS) as following: 0, normal; 1-2 = 1, no MASH; 3-4 = 2, no MASH; 5-6 = 3, mild MASH and 7-8 = 4, severe MASH. Patients with a MAS score 1 or 2 were designed as steatosis and those with 3 or 4 as suffering from MASH, respectively.

Table S2. Expression of IL-32 and CCL20 mRNA and their non-parametric correlation analysis with histologically proven inflammation and obesity.

	Inflammation			Obesity		
PNPLA3		(CC)	(CG/GG)		(CC)	(CG/GG)
n	0 = 69<br>1 = 20<br>2 = 22<br>3 = 10	0 = 33<br>1 = 8<br>2 = 8<br>3 = 4	0 = 35<br>1 = 11<br>2 = 12<br>3 = 6	0 = 40<br>1 = 44<br>2 = 27<br>3 = 10	0 = 22<br>1 = 17<br>2 = 10<br>3 = 4	0 = 18<br>1 = 27<br>2 = 14<br>3 = 5
IL-32	**0.197** *	0.192	0.205	**0.190** *	0.157	0.181
CCL20	**0.368** **	**0.375** **	**0.364** **	**0.183** *	**0.275** *	0.020

Spearman-correlation coefficient with * $p < 0.05$, ** $p < 0.01$

Inflammation: 0: no foci, 1: <2 foci, 2: 2-4 foci, 3: > 4 foci

Obesity: 0: BMI ≤ 25, 1: BMI > 25, 2: BMI ≥ 30, 3: BMI ≥ 35 kg/m^2.

Supplementary References

1. Kaur S, Rawal P, Siddiqui H, Rohilla S, Sharma S, Tripathi DM, et al. Increased Expression of RUNX1 in Liver Correlates with NASH Activity Score in Patients with Non-Alcoholic Steatohepatitis (NASH). Cells. 2019;8(10). Epub 2019/10/23. doi: 10.3390/cells8101277.
2. Weiss TS, Lupke M, Ibrahim S, Buechler C, Lorenz J, Ruemmele P, et al. Attenuated lipotoxicity and apoptosis is linked to exogenous and endogenous augmenter of liver regeneration by different pathways. PLoS One. 2017;12(9):e0184282. Epub 2017/09/07. doi: 10.1371/journal.pone.0184282.

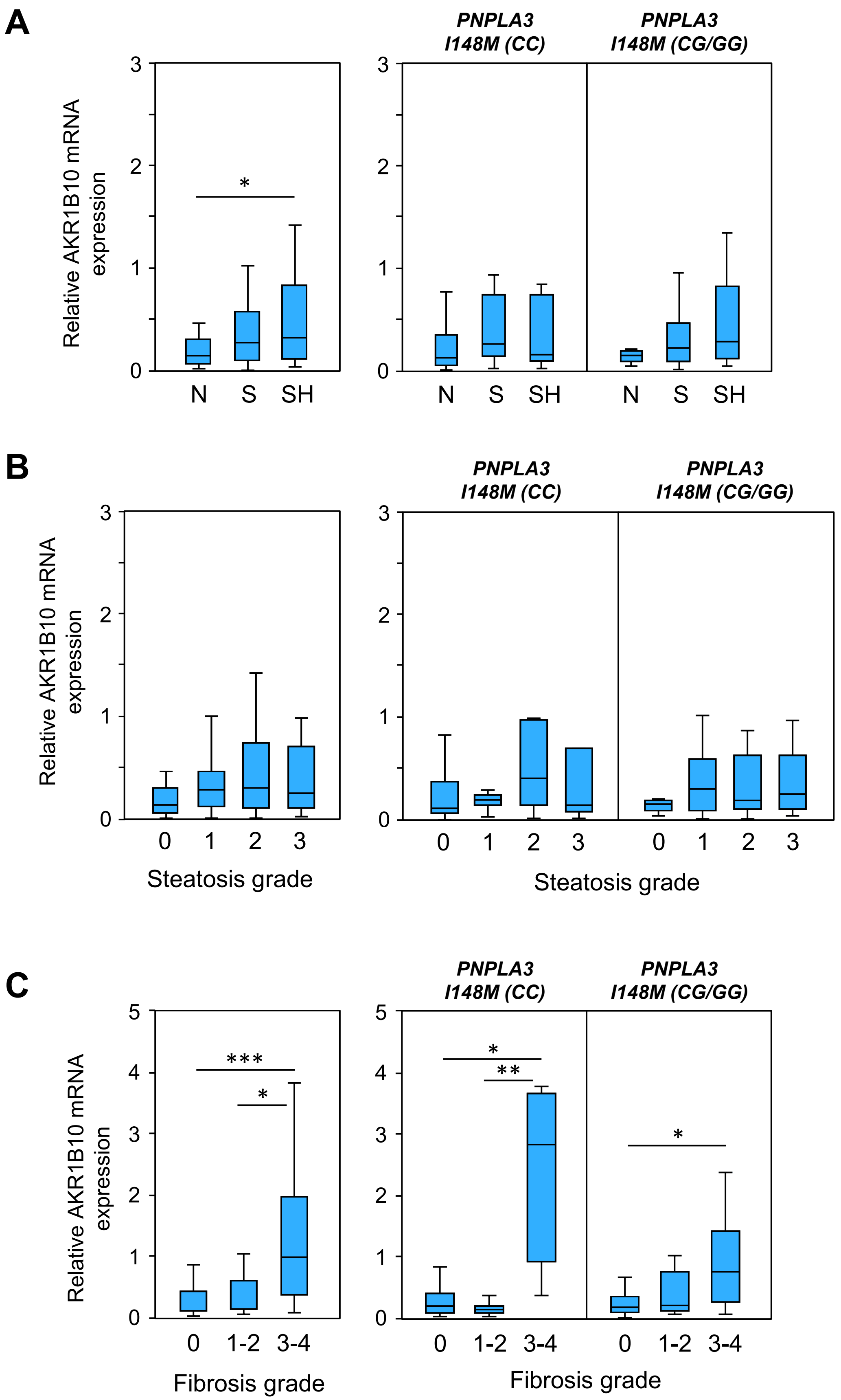

Figure S1. Expression of AKR1B10 in liver samples from patients with MASLD. A) mRNA expression was analyzed in liver tissue with MASH (SH, n = 43), hepatic steatosis (S, n = 46) and normal liver tissue (N, n = 32) by qRT-PCR and stratified by the presence of the PNPLA3 I148M variant. PNPLA3 (CG/GG), variant carrier N = 11 (10/1), S = 30 (27/3), SH = 24 (19/5) versus CC, non-carrier N = 21, S = 16, SH = 16 are shown. B) mRNA levels were analyzed in patients with different histopathologic steatosis grade (0 = 32, 1 = 25, 2 = 44, 3 = 21) and stratified by the presence of the PNPLA3 I148M variant. PNPLA3 (CG/GG), variant carrier 0 = 11 (10/1), 1 = 17 (17/0), 2 = 27 (24/3), 3 = 10 (5/5) versus CC, non-carrier 0 = 21, 1 = 7, 2 = 16, 3 = 9 are shown. C) mRNA levels were analyzed in patients with different histopathologic fibrosis grade (0 = 76, 1-2 = 27, 3-4 = 16) and stratified by the presence of the PNPLA3 I148M variant. PNPLA3 (CG/GG), variant 0 = 38 (34/4), 1-2 = 15 (11/4), 3-4 = 10 (9/1) versus CC, non-carrier (0 = 37, 1-2 = 10, 3-4 = 5) are shown. YWHAZ mRNA expression was determined for normalization. Statistical differences were analyzed by Kruskal-Wallis Test with post-hoc Bonferroni correction. * $p < 0.05$, ** $p < 0.01$.

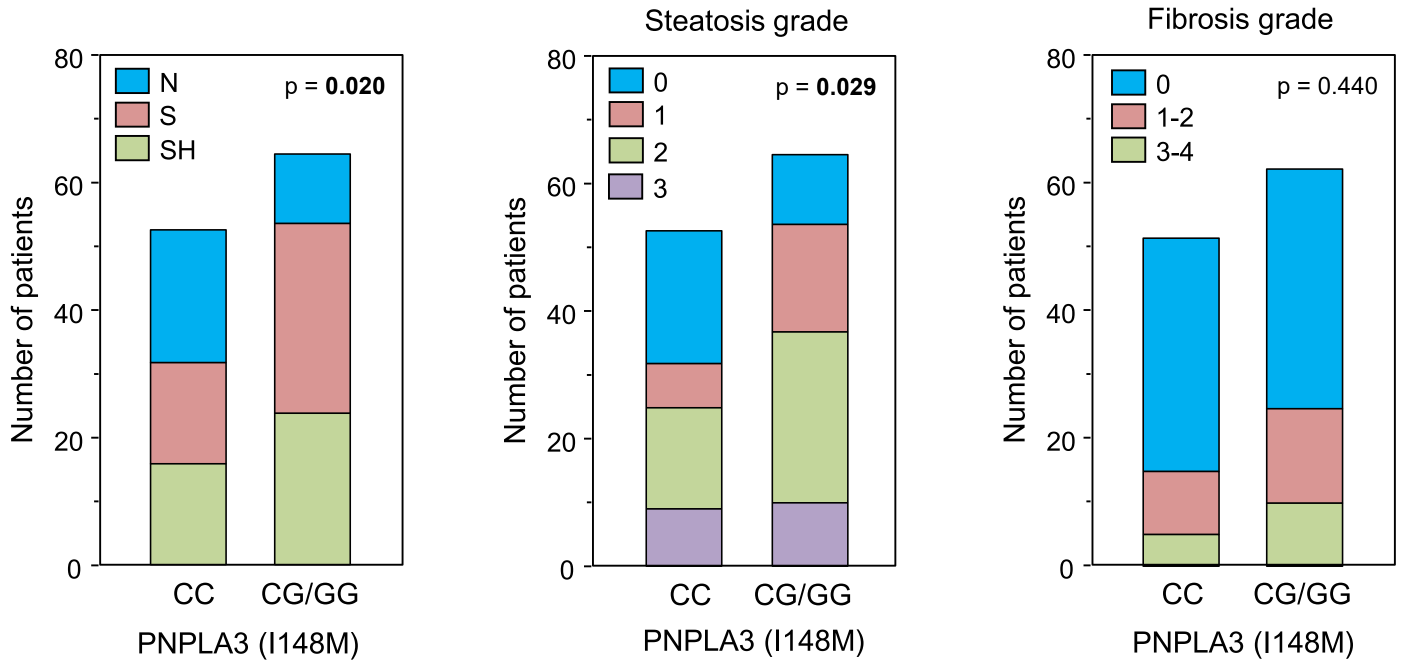

Figure S2. PNPLA3 gene variant carrier stratification of liver samples analyzed in the study. Number of patients (cases) within normal liver (N), steatosis (S) and steatohepatitis (SH) as well as histopathological steatosis grade and fibrosis grade were plotted for PNPLA3 (I148M) variant non carrier (CC) and carrier (CG/GG). Statistical differences were analyzed by cross-classified table with Pearson-Chi-Quadrat method. * $p < 0.05$.

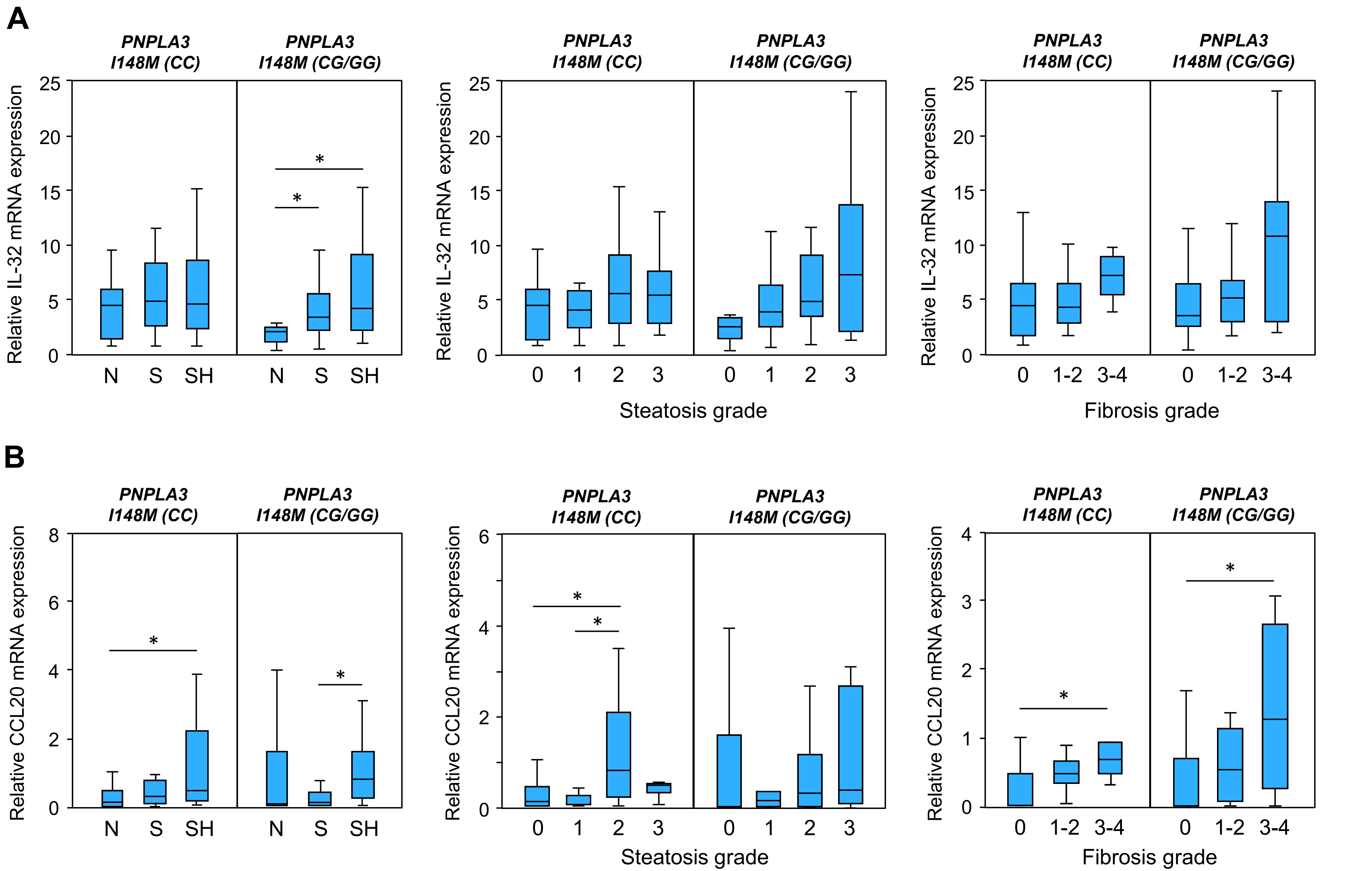

Figure S3. Expression of IL-32 and CCL20 mRNA in patients with MASH (SH), hepatic steatosis (S) and normal liver tissue (N) stratified by the presence of the PNPLA3 I148M variant. A) IL-32 and B) CCL20 mRNA expression were analyzed regarding their disease severity (N, S, SH), histopathologic steatosis grade and fibrosis grade. N, S, SH: PNPLA3 (CG/GG), variant carrier N = 11 (10/1), S = 30 (27/3), SH = 24 (19/5) versus CC, non-carrier N = 21, S = 16, SH = 16; steatosis grade: PNPLA3 (CG/GG), variant carrier 0 = 11 (10/1), 1 = 17 (17/0), 2 = 27 (24/3), 3 = 10 (5/5) versus CC, non-carrier 0 = 21, 1 = 7, 2 = 16, 3 = 9; fibrosis grade: PNPLA3 (CG/GG), variant 0 = 38 (34/4), 1-2 = 15 (11/4), 3-4 = 10 (9/1) versus CC, non-carrier 0 = 37, 1-2 = 10, 3-4 = 5. YWHAZ mRNA expression was determined for normalization and statistical differences were analyzed by Kruskal-Wallis Test with post-hoc Bonferroni correction. * $p < 0.05$ were considered significant.
